# Tracing dynamic expansion of human NK-cell subsets by high-resolution analysis of KIR repertoires and cellular differentiation

**DOI:** 10.1002/eji.201444464

**Published:** 2014-05-07

**Authors:** Vivien Béziat, James Traherne, Jenny-Ann Malmberg, Martin A Ivarsson, Niklas K Björkström, Christelle Retière, Hans-Gustaf Ljunggren, Jakob Michaëlsson, John Trowsdale, Karl-Johan Malmberg

**Affiliations:** 1 Department of Medicine, Center for Infectious Medicine, Karolinska InstitutetStockholm, Sweden; 2 Department of Pathology, Cambridge Institute for Medical ResearchCambridge, United Kingdom; 3 Department of Medicine, Liver Immunology Laboratory, Karolinska InstitutetStockholm, Sweden; 4 Établissement Français du Sang, Université de NantesNantes, France; 5 Institute for Cancer Research, Oslo University HospitalOslo, Norway; 6 Institute of Clinical Medicine, University of OsloOslo, Norway

**Keywords:** Differentiation, FACS, Killer cell immunoglobulin-like receptor, NKG2C, NK cells, repertoire

Natural killer (NK) cells are key cellular components of the innate immune system that act at the interface between innate and adaptive immune responses 1. An increasing body of evidence shows that specific clones of NK cells may be expanded in vivo under the influence of viruses such as human cytomegalovirus (CMV) 2,3. These adaptive-like NK-cell responses have been proposed to represent a human counterpart to the NK-cell memory responses observed in mice 4, and seem to be driven by activating receptors, including NKG2C and activating killer cell immunoglobulin-like receptors (KIRs) 2,5,6. So far, clonal-like expansion of specific NK-cell subsets has been documented mostly in the context of primary CMV infection, or conditions that are linked to a clinical or subclinical reactivation of CMV 2,3,6–9. Even so, there is an increasing interest in mapping adaptive-like NK-cell responses in other acute or chronic infections as well as in cancer.

The dynamic expansion and functional tuning (education) of NK cells are modulated by activating and inhibitory KIRs interacting with polymorphic determinants (KIR ligands) on HLA class I molecules 10,11. Expression of distinct KIRs at the cell surface on T and NK cells is stochastic and is influenced by variations in gene copy number and sequence 12–15. Therefore, analysis of KIR repertoires on populations of T and NK cells by flow cytometry across a wide range of *HLA* and *KIR* backgrounds represents a significant challenge. Protocols for such analysis must overcome intrinsic limitations in available reagents, cross-reactivity of monoclonal antibodies (mAbs) due to the high degree of similarity between *KIR* gene products and unexpected staining patterns resulting from *KIR* gene polymorphisms 16,17. Here, we describe recently developed staining procedures and an optimized workflow to accurately analyze the human KIRome using flow cytometry and the implementation of this protocol in the evaluation of adaptive-like NK-cell responses.

Our recent analysis of KIR expression on NK cells in 204 healthy individuals in large part employed the strategy outlined below. That study first unveiled a significant proportion of rare staining patterns that precluded a standard down-stream analysis by Boolean gating in the software 2. Genetic testing revealed that most of these patterns were caused by the previously described unusual binding patterns of specific anti-KIR antibodies to allelic variants of KIR2DL3, such as KIR2DL3*005 and KIR2DL3*015 17. To accommodate these atypical expression patterns in the analysis of NK-cell repertoires, a refined 15-color flow cytometry panel and a flowchart with sequential quality controls (QCs) was developed (Fig.1 and Supporting Information Fig. 1). This system enabled us to verify the presence or absence of specific KIRs at the cell surface. As shown in the flow chart (Supporting Information Fig. 1), the outlined strategy can be implemented in the absence of high-resolution *KIR* genotyping; however, typing all individuals for their *KIR* gene content is highly recommended.

**Figure 1:**
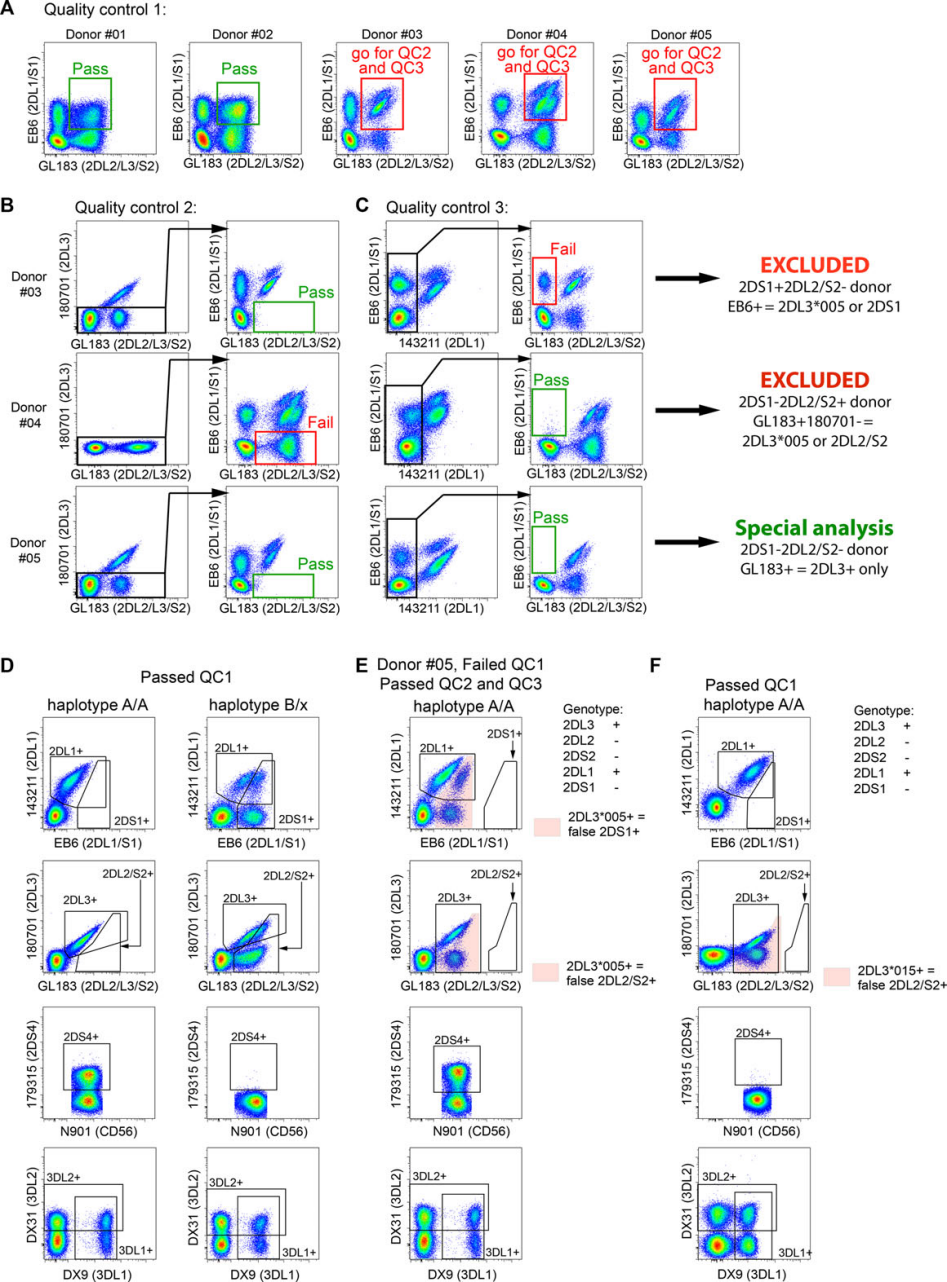
Identification of NK-cell subsets and quality controls (QCs). (A) QC1: Flow cytometry-based identification of KIR2DL3 × 005^+^ donors. The GL183 versus EB6 flow cytometry profiles of donors with the KIR2DL3*005 allele were compared with donors displaying common KIR2DL3 alleles after gating on CD3^−^CD56^dim^ NK-cells. (B and C) Donors displaying a diagonal staining in QC1, must undergo QC2 and QC3 to identify and exclude donors expressing KIR2DL3*005 in combination with KIR2DL2/S2 or KIR2DS1. KIR2DS1^+^ and KIR2DL2/S2^+^ donors have EB6^+^GL183^−^143211^−^ or EB6^−^GL183^+^180701^−^ subsets, respectively. (D) Examples of normal Boolean gating procedures in one conventional *KIR* haplotype A/A donor and one conventional haplotype B/X donor. (E) Boolean gating strategy for a KIR2DL3*005^+^ donor lacking *KIR2DS1*, *KIR2DS2*, and *KIR2DL2*. (F) Gating strategy for one representative KIR2DL3*015^+^ donor. The KIR2DL3*015 allele gives an unusual KIR staining pattern (GL183^+^180701^−^EB6^−^143211^−^) and appears as a false-positive population in the KIR2DL2/S2 gate. Data in A–F are representative of 260 donors acquired during more than 20 independent experiments.

The first QC is based on combining the anti-KIR antibodies EB6 (anti-KIR2DL1/S1) and GL183 (anti-KIR2DL2/L3/S2). In Figure1A, five typical KIR expression patterns are shown. Whereas donors #1 and #2 display normal expression patterns and can be subjected to a standard Boolean gating strategy, donors #3, #4, and #5 exhibit a diagonal staining pattern as well as multiple populations in the double-positive gate, thus requiring a further QC check before downstream analysis can be undertaken (Fig.1B and C). Indeed, this staining pattern is the hallmark of the expressed KIR2DL3*005 allele and results in a false-positive signal in the KIR2DL2/S2 and KIR2DS1 gates, respectively (Fig.1D and E) 17. As a reference material for correct interpretation of staining patterns, we provide the staining and relevant genotypes for 54 healthy donors (Supporting Information Fig. 2). In the absence of *KIR* genotyping, the decision to include donors with peculiar staining patterns in downstream KIR repertoire analysis can be based on the results of QC2 and QC3. These QCs allow for identification and exclusion of donors with KIR2DL3*005^+^ NK cells co-expressing KIR2DL2/S2 and/or KIR2DS1, since the latter KIRs cannot be distinguished from KIR2DL3*005 with currently available mAbs. For donors passing QC1, the Boolean gating is straightforward as exemplified for one typical haplotype A/A and one typical haplotype B/X donor (Fig.1D). However, KIR2DL3*005^+^ donors without *KIR2DL2/S2* and KIR2DS1 genes (e.g., Group A *KIR* haplotype homozygotes) can still be included in a modified Boolean gating algorithm, as outlined in Fig.1E, since GL183 and 143211 stain solely for KIR2DL3 and KIR2DL1, respectively, in such donors. Additional high-resolution *KIR* genotyping allows identification of KIR2DL3*015^+^ individuals whose expression of KIR2DL3*015 display an unusual KIR staining pattern (GL183^+^180701^−^EB6^−^143211^−^), and appears as a false-positive in the KIR2DL2/S2 gate (Fig.1F) 17. Of note, as for KIR2DL3*005^+^, donors with KIR2DL2/S2^+^KIR2DL3*015^+^ subsets cannot be included in downstream Boolean gating strategies.

Once the Boolean gating is set it is possible to analyze the expression of *n* KIRs and the 2*^n^* combinations thereof, allowing analysis of the KIR repertoires in cohorts of patients or healthy donors. Using this strategy, we recently found that 40% of healthy CMV seropositive blood donors displayed a profound skewing of their KIR repertoires with clonal-like expansions of KIR^+^ NK cells 2. Such expansions display significant alterations in NK-cell phenotype, including increased expression of CD57 and LILRB1, loss of Siglec-7, CD7, NKp30, FcεR1γ, and CD161 2,7,18,19. To identify a skewing of the KIR repertoire, and expansions of discrete KIR-expressing NK-cell subsets, two alternative, and not mutually exclusive, strategies can be applied: (i) a statistical approach identifying donors with KIR repertoires that fall outside the normal distribution; and (ii) a phenotypic approach, identifying donors with alterations of cell surface receptors. Below we illustrate the advantages and disadvantages of the two methods by analyzing the KIR repertoire and cell surface phenotype in an additional cohort of 60 healthy blood donors.

Using the Boolean gating strategy described above, 128 subsets of KIR-expressing NK cells from 60 donors were generated, and their relative frequencies among NKG2A^+^, NKG2C^+^NKG2A^−^, and NKG2C^−^NKG2A^−^ NK cells were plotted (Fig.2A). Of note, NKG2A^+^NKG2C^+^ cells, representing on average 1.6% of all NK cells, were included in the global analysis of NKG2A^+^ NK cells. Next we used the Chauvenet's criterion to identify the statistical outliers in each of the NK-cell subsets (Fig.2A). The values falling outside of the normal distribution identify donors that have a skewed KIR repertoire, and likely contain clonal-like expansions. By using the alternative, phenotypic approach, we tested whether the identified outliers represented clonal-like expansions. The KIR2DL2/S2^+^ NK cells in donor #018 identified by the statistical approach expressed low levels of NKp30 and high levels of CD57, consistent with a differentiated phenotype (Fig.2B). In contrast, the outlier expressing KIR3DL1 in donor #034 expressed normal levels of NKp30 (Fig.2C), suggesting that this was a false-positive outlier. Thus, the statistical approach sometimes results in identification of false-positive outliers. In order to optimize the statistical approach, additional criteria can be implemented, including thresholds for the proportion of the expanded phenotype relative to all NK cells or the relevant NK-cell subsets (e.g. NKG2A^+^, NKG2C^−^NKG2A^−^, or NKG2C^+^NKG2A^−^). Figure2E depicts the frequency of false-positive (type I errors) and false-negative (type II errors) expansions using different thresholds for required frequency among total NK cells and NK-cell subpopulations. The phenotypic approach was used to determine the frequency of donors with clonal-like NK-cell expansions, and to determine the frequency of type I and II errors generated by the statistical method. With a very low threshold, the statistical approach included many false-positive subsets (blue) with relatively high KIR frequency but with a normal phenotype. On the other hand, high thresholds resulted in significant type II errors, that is, failure to detect some NK-cell expansions with an altered phenotype. Thus, although profound deviations in KIR expression are highly specific for clonal-like NK-cell expansions, the statistical approach may be too insensitive to pick up more subtle changes in the NK-cell repertoire, particularly in smaller cohorts. Examination and quantification of clonal-like NK-cell expansions are thus most robustly performed by swapping the order of the analysis: First by screening for phenotypic changes, then by applying in-depth characterization of KIR expression within the clonal phenotypes (Supporting Information Fig. 1). In such a reversed approach, further down-stream analysis of the clonal phenotypes can be undertaken to resolve the expression of activating KIRs, as illustrated by the use of 1F12 antibody in our panel, which allows the detection of KIR2DS2^+^ cells (Supporting Information Fig. 3) 20. Selected phenotypic/differentiation markers can also be replaced to resolve the expression of 3DS1 and 2DS5, as previously described 16,21. The choice of NKp30 and CD57 as markers for positive identification of expanded and differentiated cell populations was based on our analysis of NK-cell repertoires in 204 healthy donors 2. However, other combinations of differentiation markers may be considered, in particular for expansions with a less clear loss of NKp30 and/or normal expression of CD57. As the phenotypic approach uses simultaneous staining of multiple KIRs, NKG2A, NKG2C, and markers of NK-cell differentiation, it requires 13–15 color flow cytometry. The statistical approach can thus be useful when KIR stainings are available in the absence of markers of NK-cell differentiation, provided that the studied cohort is large enough (*n* ≈ 40) and that sufficiently high thresholds for frequency of total NK cells and NK-cell subsets are used.

**Figure 2:**
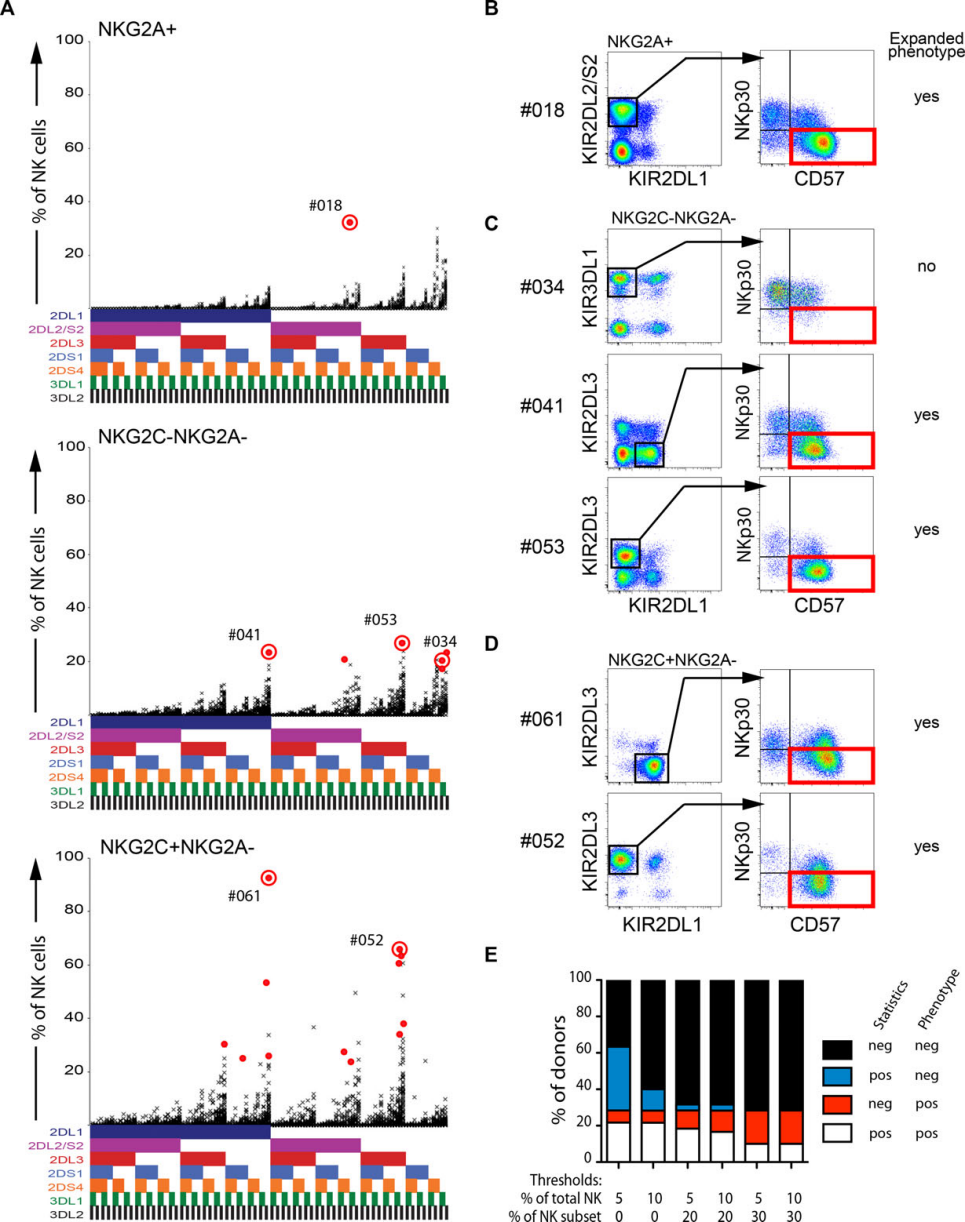
Approaches for detection of adaptive-like NK-cell responses. (A) Statistical approach. The frequency of the NK-cell subsets expressing the seven analyzed KIRs and the 128 possible combinations thereof in 60 healthy donors is plotted in a single graph. The presence of one KIR in a combination is represented by a color code below the graph: 2DL1 (dark blue), 2DL2/S2 (purple), 2DL3 (red), 2DS1 (light blue), 2DS4 (orange), 3DL1 (green), and 3DL2 (black). The analysis is displayed for NKG2A^+^, NKG2A^−^NKG2C^−^, and NKG2A^−^NKG2C^+^ subsets. Examples of statistical outliers, as identified by Chauvenet's criterion, are highlighted in red. (B–D) Phenotypic approach. Detection of NK cells that have expanded and differentiated as defined by their differentiated NKp30^lo^CD57^+^ phenotype. Data in B–D are representative of 60 donors acquired during six independent experiments. The expansion observed in donor #18 was NKG2A^+^NKG2C^+^. (E) Comparison of the number of false-positive and verified expansions by combining the statistical approach with phenotypic verification. Thresholds of statistical outliers was set to >5% or >10% of the total NK cells and >0%, >20% or >30% of NKG2A^+^, NKG2C^+^NKG2A^−^, or NKG2C^−^NKG2A^−^ NK-cell subsets.

In conclusion, we have here outlined an algorithm for stepwise analysis of KIR expression patterns using a combination of commercially available KIR antibodies. The algorithm can be employed to accurately determine human KIR repertoires via single-cell analysis platforms such as flow cytometry or CyTOF. By combining KIR repertoire analysis with assessment of differentiation states, the proposed algorithm can be used to determine adaptive-like NK-cell responses in various clinical conditions.

## Abbreviations

KIR: killer cell immunoglobulin-like receptor
QC: quality control

## Supporting Information

The detailed *Materials and methods* for Technical comments are available online in the Supporting information
