# Supplementary material for: Tracing dynamic expansion of human NK-cell subsets by high-resolution analysis of KIR repertoires and cellular differentiation
Source: Eur J Immunol. 2014 May 7;44(7):2192–6. doi: 10.1002/eji.201444464 (PMC4282447; doi:10.1002/eji.201444464)
Supplement: Supplementary file 1 — Supplementary [file eji0044-2192-SD1.pdf]

European Journal of Immunology

Supporting Information
for
DOI 10.1002/eji.201444464

Vivien Béziat, James Traherne, Jenny-Ann Malmberg, Martin A. Ivarsson, Niklas K. Björkström, Christelle Retière, Hans-Gustaf Ljunggren, Jakob Michaëlsson, John Trowsdale and Karl-Johan Malmberg

Tracing dynamic expansion of human NK-cell subsets by high-resolution analysis of KIR repertoires and cellular differentiation

© 2014 The Authors. *European Journal of Immunology* published by Wiley-VCH Verlag GmbH & Co. KGaA, Weinheim.

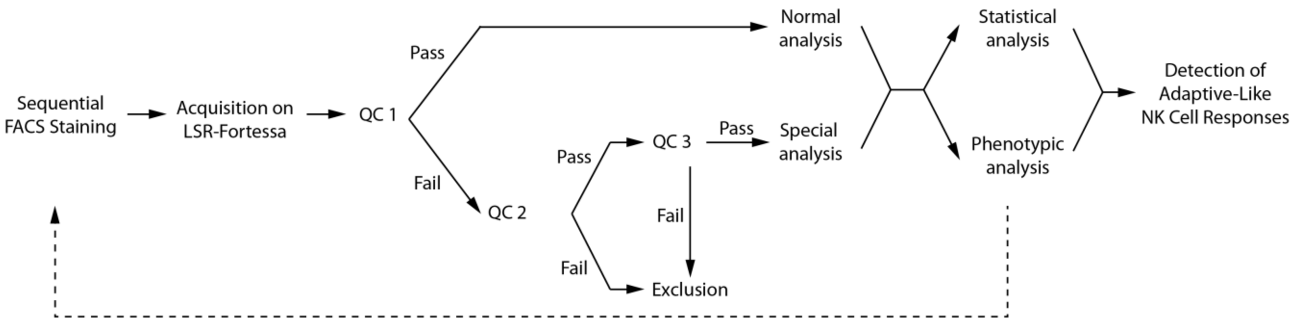

Supporting Information Figure 1. Schematic workflow for KIR repertoire analysis
Sequential staining for KIR expression followed by statistical assessment of outliers. KIR repertoire analysis is combined with phenotypic assessment of differentiation states. In a reversed approach, the KIR repertoire analysis is performed downstream of identification of subsets with terminally differentiated states (dashed arrow).

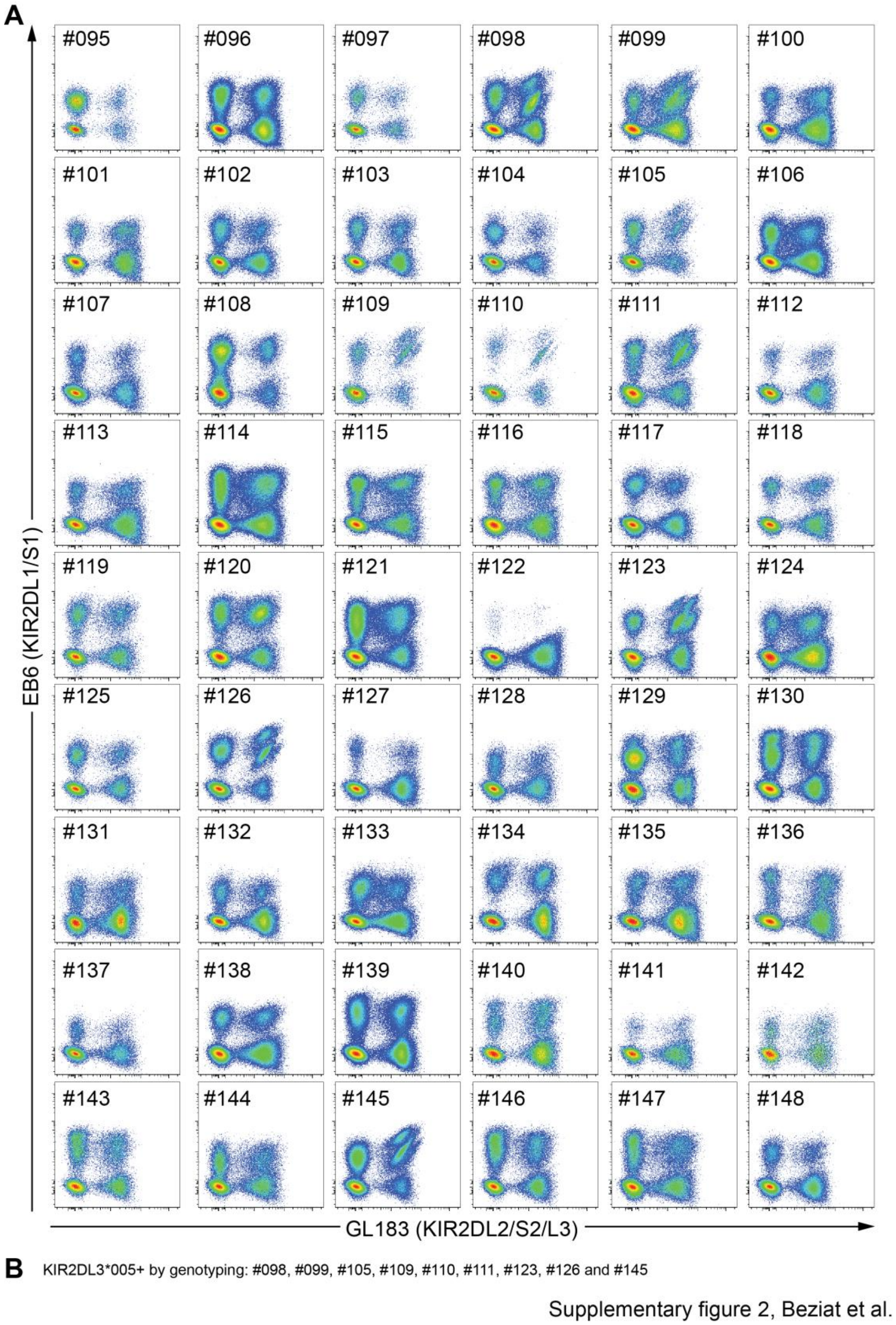

Supporting Information Figure 2. Detection of the diagonal staining typical for *KIR2DL3*005* genotypes. The GL183 versus EB6 stainings of 54 donors from a previously characterized cohort are depicted [1]. Donors with a KIR2DL3*005 allele display a single or multiple diagonal staining in the GL183+EB6+ quadrant. The donors confirmed to be KIR2DL3*005+ by genotyping are: #098, #099, #105, #109, #110, #111, #123, #126, and #145.

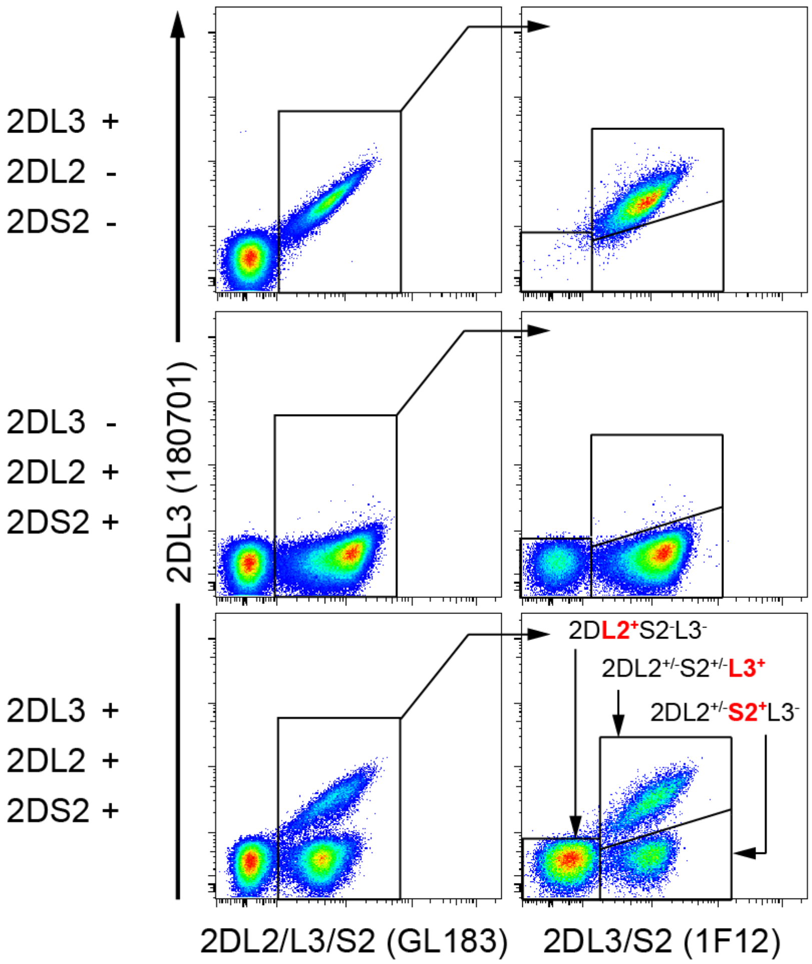

Supporting Information Figure 3. Combinatorial staining for detection of KIR2DL2 and KIR2DS2. Resolution of KIR2DL2, KIR2DS2 and KIR2DL3 using an anti KIR2DL3/S2 antibody (1F12). Three representative donors with different gene content are depicted.

Supporting Information Table 1. Antibody specifications and laser configuration

Specificity	Clone	Color	Supplier	Laser excitation (nm)	Filter	Mix 1	Mix 2	Mix 3
KIR2DL3	180701	FITC	R&D	488	530/30	X		
KIR2DL1	143211	APC	R&D	639	670/30	X		
NKG2C	134591	AF700	R&D	639	730/45	X		
NKG2A	Z199	APC-AF750	Beckman Coulter[1]	639	780/60	X		
KIR3DL1	DX9	BV421	Biolegend	405	450/50	X		
CD14	M5E2	Horizon V500	Becton Dickinson	405	525/50	X		
CD19	HIB19	Horizon V500	Becton Dickinson	405	525/50	X		
Dead cell marker aqua	-	-	Invitrogen	405	525/50			X
KIR2DS4	179315	Qdot-585	In-house conjugation[2]	405	585/42	X		
KIR2DS2/L3	1F12	Biotin	In-house conjugation[3]	-	-	X		
Streptavidin	-	Qdot-605	Invitrogen	405	610/20			X
CD57	TB01	Unconjugated	eBioscience	-	-	X		
Anti-IgM	-	eFluor-650	eBioscience	405	670/30			X
KIR3DL2	DX31	BV711	Biolegend[4]	405	710/50	X		
NKp30	AF29-4D12	PE	Miltenyi	561	586/15	X		
CD56	N901	ECD	Beckman Coulter	561	610/20	X		
CD3	UCHT1	PE-Cy5	Beckman Coulter	561	661/20	X		
KIR2DL2/L3/S2	GL183	PE-Cy5.5	Beckman Coulter	561	710/50		X	
KIR2DL1/S1	EB6	PE-Cy7	Beckman Coulter	561	780/60		X	

[1]NKG2A-APC-AF750 was a custom order from Beckman Coulter.
[2]KIR2DS4 (179315, R&D systems) was conjugated using a Qdot-585 conjugation kit (Q22011MP) from Invitrogen.
[3]KIR2DL3/S2 (1F12), was provided by Dr. C. Retière and conjugated using a biotinylation kit (FluoReporter Mini-biotin-XX Protein Labeling Kit, Invitrogen) after overnight dialysis in PBS-1x using a 10-30kd cassette (Thermo Scientific).
[4]KIR3DL2-BV711 was a custom order from Biolegend upon supply of purified DX31.

Supporting Information Material and Methods

FACS staining

This study was approved by the regional ethics committee in Stockholm, Sweden. For each donor, $2x10^6$ fresh cells were plated in a 96 V-bottom well plate, washed with FACS buffer (PBS-1x, 2mM EDTA, 2% FCS) and stained with 50µL of Mix 1 (Supporting Information Table 1) for 20 minutes at room temperature. 50µL of Mix 2 (Supporting Information Table 1) was added in each well without washing for 10 additional minutes at room temperature. Next, cells were washed twice with FACS buffer and stained for 30 minutes with 50µL of Mix 3 (Supporting Information Table 1) at room temperature. After this last staining step, cells were washed once, fixed with Fixation Permeabilization Buffer Set (Ebioscience) for 20 minutes. Finally, the fixed cells were washed once with FACS buffer, resuspended in 200µL FACS buffer and acquired on a LSR-Fortessa cell analyzer (BD Biosciences). Data was analyzed with FlowJo software version 9.4.7. The lasers and filters used for detection of each parameter are indicated in Supporting Information Table 1.

KIR genotyping

Global *KIR* genotyping was performed using a recently described high-throughput technology called qKAT[2]. The *2DL3*005* genotype was determined in two steps. Firstly, PCR amplification with the following pair of sequence-specific primers (PCR-SSP) sited within exon 4, which specifically amplify only three 2DL3 alleles (*004, *005 and *010); forward 5′-GTCCACAGAAAACCTTCCCTCAG-3′ and reverse 5′- GGTGCAAAGTGTCCTTAA ACTTCCTT-3′. Internal control primers that amplify a 796-bp fragment of the third intron of DRB1 were also included in each PCR to validate proper amplifications. Genomic DNA samples that were positive by the above PCR-SSP were sequenced over a region that included two SNP sites in exon 7 that distinguish *2DL3*005* from *2DL3*004* and *2DL3*010*, and a third nucleotide site in exon 7 that confirmed the amplifications were from 2DL3. The forward and reverse PCR primers and sequencing primer were 5′-GTGTCTCCTCTTCTTCCAGGTAATC-3′, 5′-GCAGGCTCTTGGTCCATTACTA-3′, 5′-GGGACCATCCTGTCTGTGAG-3′, respectively. The forward primer is sited at the intron 6-exon 7 boundary, the reverse primer is sited in exon 8 and the sequencing primer is sited within intron 7. The penultimate 3′ nucleotide of the SSPs for both reactions is a mismatch to all KIR gene sequences to prevent nonspecific priming. Amplifications were performed in a volume of 10 μl containing 1x Biomix clear PCR mix (Bioline), 500 nM primer and 10 ng of DNA. Cycling was performed as follows: 10 mins at 95°C; 3 cycles of 96°C for 24 seconds, 65°C for 45 seconds and 72°C for 45 seconds; 30 cycles of 96°C for 25seconds, 62°C for 45 seconds and 72°C for 45 seconds; 3 cycles of 96°C for 25 seconds, 55°C for 1 min and 72°C for 2 mins; 72 °C for 10 mins. PCR products were electrophoresed in 2% agarose gels containing ethidium bromide, and predicted size products were visualized under UV light.

The *KIR2DL3*015* genotype was determined using polymerase chain reaction amplifications with the following pair of M13-tailed sequence-specific primers (PCR-SSP): forward 5′-CCCCTGGTGATCGTGGT-3′ and reverse 5′-CGGACACTCTCACCTGTAATG-3′ followed by sequencing the product using M13R sequencing primer. This assay was only used on KIR A haplotype homozygote samples.

References

1 Beziat, V., Traherne, J. A., Liu, L. L., Jayaraman, J., Enqvist, M., Larsson, S., Trowsdale, J. and Malmberg, K. J., Influence of KIR gene copy number on natural killer cell education. *Blood* 2013. 121: 4703-4707.

2 Jiang, W., Johnson, C., Jayaraman, J., Simecek, N., Noble, J., Moffatt, M. F., Cookson, W. O., Trowsdale, J. and Traherne, J. A., Copy number variation leads to considerable diversity for B but not A haplotypes of the human KIR genes encoding NK cell receptors. *Genome Res* 2012.
