# Supplementary material for: Tracing dynamic expansion of human NK-cell subsets by high-resolution analysis of KIR repertoires and cellular differentiation
Source: Eur J Immunol. 2014 May 7;44(7):2192–6. doi: 10.1002/eji.201444464 (PMC4282447; doi:10.1002/eji.201444464)
Supplement: Supplementary file 2 — Supplementary [file eji0044-2192-SD2.pdf]

European Journal of Immunology

Peer review correspondence

Tracing dynamic expansion of human NK-cell subsets by high-resolution analysis of KIR repertoires and cellular differentiation

Vivien Béziat, James Traherne, Jenny-Ann Malmberg, Martin A. Ivarsson, Niklas K. Björkström, Christelle Retière, Hans-Gustaf Ljunggren, Jakob Michaëlsson, John Trowsdale and Karl-Johan Malmberg

Correspondence: Prof. Karl-Johan Malmberg, Institute of Clinical Medicine, University of Oslo, Norway

---

Review Timeline:	Submission date:	13 January 2014
	First Editorial decision:	11 February 2014
	First Revision received:	27 February 2014
	Accepted:	04 April 2014

Handling Executive Committee member: Prof. Marco Colonna

Please note that the correspondence below does not include the standard editorial instructions regarding preparation and submission of revised manuscripts, only the scientific revisions requested and addressed.

First Editorial Decision – 11 February 2014

Dear Dr. Malmberg,

Manuscript ID eji.201444464 entitled "Tracing dynamic expansion of NK cell subsets by high resolution analysis of killer cell immunoglobulin-like receptor repertoires" which you submitted to the European Journal of Immunology has been reviewed. The comments of the referees are included at the bottom of this letter.

A revised version of your manuscript that takes into account the comments of the referees will be reconsidered for publication.

You should also pay close attention to the editorial comments included below.

Please note that submitting a revision of your manuscript does not guarantee eventual acceptance, and that your revision will be re-reviewed by the referees before a decision is rendered.

If the revision of the paper is expected to take more than three months, please inform the editorial office. Revisions taking longer than six months may be assessed by new referees to ensure the relevance and timeliness of the data.

Once again, thank you for submitting your manuscript to European Journal of Immunology and we look forward to receiving your revision.

Yours sincerely,
Karen Chu

Peer review correspondence

European Journal of Immunology

On behalf of Prof. Marco Colonna

Dr. Karen Chu
Editorial Office
European Journal of Immunology
e-mail: ejied@wiley.com
www.eji-journal.eu

****

Reviewer: 1

Comments to the Author
Beziat et al. present a technical comment that builds on their previous studies to provide an algorithm to analyze KIR expression patterns. In addition, they present a combined phenotypic/statistical method to identify outliers in terms of expression patterns, that could be consistent with expansions of adaptive-like NK cells. Overall, the manuscript is very clearly written and the data is beautiful and presented in a convincing and straightforward manner. The findings, while not particularly novel in light of their previous work, are solid and provide a useful tool for the NK cell field. One overarching concern for its broad impact is the fact that relatively few centers regularly perform 13-15 color flow cytometry with the skill required to clearly distinguish “multiple” or “diagonal” vs. “single” populations, particularly for less skilled operators where compensation could be more of an issue. While CyTOF may eventually provide a solution to the quality and compensation issues, the authors may want to make clear that it is possible but not a certainty.

Specific comments:

With regard to Quality Control 1, I have some concern that “Pass” vs. “going on to QC2 and QC3” could be somewhat subjective—particularly if the compensation is an issue. Would it be possible to test how reliable this is (ie by having blinded individuals select whether it passes or not)? Could the authors explain the ramifications of a false call at that stage and whether that should be addressed in their pipeline if false calls are likely?

In terms of determining the false positive and negative calls by the statistical method, can the authors please explain more clearly how confident they are with their phenotypic definition? Is there some cutoff in NKp30 downregulation that makes them feel confident that they can call an adaptive-like cell, given the heterogeneity in the population? While this may be addressed in earlier papers, since it’s being used essentially as a gold standard her, it would be nice to have this information for the reader.

Reviewer: 2

Comments to the Author
The technical comment entitled „Tracing dynamic expansion of NK cell subsets by high resolution analysis of killer cell immunoglobulin-like receptor repertoires“ by Beziat et al describes how the multiparameter flow cytometry analysis of KIR expression on human NK cells allows the accurate determination of the KIRome without KIR genotyping. Furthermore, they investigated how this method together with the analysis of additional previously described markers can be employed to identify clonally expanded populations in different clinical situations, even among NKG2C- NK cells. The technical workflow KIRome analysis is accurately performed and altogether the combination of the approaches described by the authors has an interesting potential to identify further receptors driving clonal expansions and to explore NK cell adaptive properties.

European Journal of Immunology

General comments:

I. The identification of the KIRome by FC without KIR genotyping is a useful method to determine accurate KIR expression and KIR allele expression. However, as technical comment more methodical details should be provided as supporting information i.e. the 15 color flow cytometry, with staining protocol, antibody-clones, conjugated fluorochromes and instrument setup (laser, detector filter combinations). Moreover, information about the software used for the Boolean gating strategy should be given. However, I think that a nice application of this technical
II. As outliers of KIR expression not always implicate clonally expanded populations, the authors suggested that other markers are necessary for identification of expansions and exclusion of false-positive results. According to the author previous publication (Beziat, 2013) and other reports (Guma, 2004, Della Chiesa, 2012), potential clonal expansions also display peculiar expression of other markers (CD161, Siglec-7, LIR-1, CD2..). Why did the authors use only CD57 and NKp30? If the aim of the technical comment is to better identify NK cell expansions, it would be very interesting to test whether the staining of additional markers would minimize the false-positive results.
III. As suggested by the dashed line in Figure S1, it would be much easier to first perform phenotypic analysis for other markers described for clonally expanded NK cell populations before starting statistical analysis of KIR expression. Since it is not the high resolution analysis of the KIRome on its own, but rather the combination of different strategies which better enables tracing human NK cell expansions, the title of the manuscript should be reconsidered.
IV. It would be much easier to follow text and figures, if the names of both, the clones and KIR genes recognized, would be consistently indicated throughout the manuscript (as depicted in Figure 1D-F, while it is missing in Figure 1A-C).
V. It is not clear how Figure S2 helps to identify clonally expanded populations, the reference in the text (pp3, line 20-24) does not clearly indicate what can be found in the figure. Moreover, the authors do not comment on Figure S2B and S2C. In line with comment I, impact and antibody conjugation should be discussed.
VI. On page 2, line 59, KIR2DL3 is written, but as indicated in Figure 2A and 2C, it should be KIR3DL1.

Minor points:
1) In Figure 2B, it would be helpful to indicate the x-axis as in 2C and 2D
2) The legend for supplementary figure 2A and D should mention the KIRotype plotted on the left side of the dot plots and how it was determined.

Reviewer: 3

Comments to the Author
In this manuscript the authors describe an approach based on the combined use of various commercially available anti-KIR mAbs, using a 15-color flow cytometry, to characterize and quantify the different NK cell subpopulations based on their KIR expression. The described protocol includes different quality controls (QCs) that provide a flowchart allowing to exclude donors characterized by a KIR repertoire that gives rise to staining misinterpretation (e.g. false KIR2DL2/S2+ or KIR2DS1+ subsets). In particular, KIR2DL3*005 and KIR2DL3*015 have been previously described to have an atypical staining pattern. Indeed, both do not react with the anti-KIR2DL3 specific mAb (i.e. 180701) and KIR2DL3*005 reacts with the anti KIR2DL1/2DS1 mAb (i.e. EB6). QC1 evaluates the presence/absence of KIR2DL3*005. QC2 allows to identify subjects characterized by a cenA/cenA genotype (i.e. KIR2DL2/2DS2 negative) whereas QC3 allows to exclude donors that are KIR2DS1 positive. Correct and unambiguous interpretation of the stainings can be assessed only for the samples that pass all QCs. As also stated by the Authors, analysis of KIR genotype is always highly recommended, indeed still unrecognized exceptions of anti-KIR mAb reactivity can possibly exist.
The proposed strategy is clear, well defined and can provide great help for studies on the characterization of NK cells expressing one or another KIR. The correct assessment of the KIR phenotype is an excellent tool for additional analysis such as evaluation of clonal expansions of NK cells or identification of markers that can characterize the different NK cell subpopulations. The authors have a solid experience and good publications in this field.

European Journal of Immunology

Minor revision are requested:
- Page 1 lines 40-41: the sentence should be modified including information on the impact of HLA class I (KIR-L) in KIR education.
- Page 2 lines 17-19: the sentence should be modified to better clarify that it is actually the presence of KIR2DL3*005, when co-expressed with KIR2DL2/S2 and/or KIR2DS1, that does not permit a further detailed analysis of the KIR repertoire.
- A table summaring all mAb used for cytofluorimetric analysis is required and may be included in the Supplementary items. This table should indicate name of the clone, reactivity, source, type of labeling. Also the various steps of incubation are important to reproduce the method.
- In the figures the + and – characters related to the various KIRs are too small and hardly visible (while these data are crucial). The use of a larger character is recommended.
- Figure 1 panel D: the choice of representative donors with A/A and B/x haplotypes would be much more appropriate to show KIR2DS4 expression in A/A instead of B/x donor.
- Page 2 lines 56-59: The identification of KIR+ NK cells in relation with data shown in Fig. 2 is unclear or wrong: KIR2DL2/S2+ (and not only KIR2DS2+) NK cells in donor #018, KIR3DL1 (and not KIR2DL3) in donor #034.
- Fig. 2 B: There is no indication of the molecule in the abscissa of both dot plots (KIR2DL1 and CD57?)
- Fig. 2: (A) the NKG2A+NKG2C+ subset has not been considered; is it included in the NKG2A+ subset? Please, briefly comment this point.
- The NKp30- CD57+ pattern has been considered as the hallmark of an expanded phenotype. Please provide the appropriate reference.
- The color code used to indicate the KIR combinations of the NK cells in each graph of figure 2 A would help the reader. Y axes may be interrupted. This would shorten the height of the panels allowing the insertion of the color code.
- Supplementary Fig. 2: two different names for mAb recognizing 2DL5 are used: UP-R1 in the figure panel D, and 5.133 in the legend. 5.133 mAb is probably incorrect. Moreover, for the detection of KIR2DS5 positive cells the authors propose the use of NKVSF1 mAb, a mAb characterized by a broad reactivity with KIR2D. Why should the authors exclude KIR2DL5 positive cells from the analysis? Is there any evidence that NKVSF1 mAb recognizes also KIR2DL5?
- A very recent paper (Della Chiesa et al. J Immunol 2014) described the expansion of NK cells expressing activating KIRs in NKG2C-/- patients experiencing CMV infection. This reference is relevant and should be added.

First Revision – authors' response – 27 February 2013

Reviewer: 1

Comments to the Author

1) Beziat et al. present a technical comment that builds on their previous studies to provide an algorithm to analyze KIR expression patterns. In addition, they present a combined phenotypic/statistical method to identify outliers in terms of expression patterns, that could be consistent with expansions of adaptive-like NK cells. Overall, the manuscript is very clearly written and the data is beautiful and presented in a convincing and straightforward manner. The findings, while not particularly novel in light of their previous work, are solid and provide a useful tool for the NK cell field. One overarching concern for its broad impact is the fact that relatively few centers regularly perform 13-15 color flow cytometry with the skill required to clearly distinguish "multiple" or "diagonal" vs. "single" populations, particularly for less skilled operators where compensation could be more of an issue. While CyTOF may eventually provide a solution to the

European Journal of Immunology

quality and compensation issues, the authors may want to make clear that it is possible but not a certainty.

Author response: We appreciate the fact that many centers may not have access to cytometers allowing 13-16 color staining. Although the present technical comment is mostly intended to facilitate KIR repertoire analysis for advanced users, the presented algorithm may be helpful also for those who are analyzing KIR expression using fewer flourochromes (combinations of 4-6 KIRs and 2-3 phenotypic markers). Such panels will not give a complete resolution of the KIR repertoire but suffice to identify donors with peculiar alleles.

Carefully designing the panel and selecting optimal fluorochromes for the key markers can overcome compensation issues. In the revised manuscript, detailed information about the clones and instrument settings are provided as supplementary information. (See also response to comment 2 below regarding the consistency of the interpretation of the diagonal staining).

Specific comments:

2) With regard to Quality Control 1, I have some concern that “Pass” vs. “going on to QC2 and QC3” could be somewhat subjective—particularly if the compensation is an issue. Would it be possible to test how reliable this is (ie by having blinded individuals select whether it passes or not)? Could the authors explain the ramifications of a false call at that stage and whether that should be addressed in their pipeline if false calls are likely?

Author response: We appreciate this concern. However, the outlined strategy is extremely robust and is implemented in the standard operating procedures for all NK cell groups at our center. So far, we have not identified one single false positive donor. It is possible that other combinations of flourochromes may yield less robust data and users will need to verify that the chosen conjugates operate as well as the ones outlined here.

To address this point we have compiled a new supplementary figure (Supplementary Figure 2) showing the staining patterns in 54 donors, out of which 9 had the 2DL3*005 allele. These donors are disclosed in the legend. Ten independent lab members, with varying experience in flow cytometry, were asked to look at Figure 1A and thereafter identify the donors with diagonal staining in Supplementary Figure 2. 10 out of 10 scored 100% in this exercise. This outcome is not mentioned in the text. However, we believe that supplementary Figure 2 will serve as a useful reference for successful implementation of the proposed strategy. See revised text on page 2.

3) In terms of determining the false positive and negative calls by the statistical method, can the authors please explain more clearly how confident they are with their phenotypic definition? Is there some cutoff in NKp30 downregulation that makes them feel confident that they can call an adaptive-like cell, given the heterogeneity in the population? While this may be addressed in earlier papers, since it’s being used

European Journal of Immunology

essentially as a gold standard her, it would be nice to have this information for the reader.

Author response: This is a very relevant point and relates to comment 2 or reviewer 2 and comment 9 of reviewer 3. NKp30 was chosen as one key marker based on our previous examination of 204 healthy donors where it was found to be highly specific (Beziat et al, Blood 2013, Figure 5B). However, some donors harbor expansions with less clear staining patterns, as exemplified in Figure 2D, Donor #61. For such expansions it may be necessary to extend the phenotypic panel and monitor additional differentiation markers. The basis for selecting NKp30 and CD57 and the potential weaknesses of the approach are now discussed on page 3.

Reviewer: 2

Comments to the Author

The technical comment entitled „Tracing dynamic expansion of NK cell subsets by high resolution analysis of killer cell immunoglobulin-like receptor repertoires“ by Beziat et al describes how the multiparameter flow cytometry analysis of KIR expression on human NK cells allows the accurate determination of the KIRome without KIR genotyping. Furthermore, they investigated how this method together with the analysis of additional previously described markers can be employed to identify clonally expanded populations in different clinical situations, even among NKG2C- NK cells. The technical workflow KIRome analysis is accurately performed and altogether the combination of the approaches described by the authors has an interesting potential to identify further receptors driving clonal expansions and to explore NK cell adaptive properties.

General comments:

1). The identification of the KIRome by FC without KIR genotyping is a useful method to determine accurate KIR expression and KIR allele expression. However, as technical comment more methodical details should be provided as supporting information i.e. the 15 color flow cytometry, with staining protocol, antibody-clones, conjugated fluorochromes and instrument setup (laser, detector filter combinations). Moreover, information about the software used for the Boolean gating strategy should be given. However, I think that a nice application of this technical

Author response: The requested information is now provided as supplementary information. Is this comment truncated since it ends in the middle of a sentence?

2). As outliers of KIR expression not always implicate clonally expanded populations, the authors suggested that other markers are necessary for identification of expansions and exclusion of false-positive

European Journal of Immunology

results. According to the author previous publication (Beziat, 2013) and other reports (Guma, 2004, Della Chiesa, 2012), potential clonal expansions also display peculiar expression of other markers (CD161, Siglec-7, LIR-1, CD2..). Why did the authors use only CD57 and NKp30? If the aim of the technical comment is to better identify NK cell expansions, it would be very interesting to test whether the staining of additional markers would minimize the false-positive results.

Author response: This comment is highly relevant and relates to comment 3 of reviewer 1 and comment 9 of reviewer 3. NKp30 was selected based on our previous observations published in Beziat et al, Blood 2013, Figure 5B). It was found to be very reliable and together with CD57, we are confident that the population we are analyzing have undergone a clonal-like expansion. Thus, the risk for false positives is minimal.

We agree that it would be very interesting to further dissect the clonal phenotype by combining KIR assessment with a broader differentiation panel. However, broadening the phenotypic panel would come at the expense of the KIR profiling. The presented algorithm could serve as a template for such modified panels but we believe it would be too complex to describe all possible variations of the differentiation markers in this technical note. However, to highlight this issue, we now discuss the choice of markers and the need for validations in cases where the NKp30/CD57 staining is indecisive. See revised text on page 3.

3) As suggested by the dashed line in Figure S1, it would be much easier to first perform phenotypic analysis for other markers described for clonally expanded NK cell populations before starting statistical analysis of KIR expression. Since it is not the high resolution analysis of the KIRome on its own, but rather the combination of different strategies which better enables tracing human NK cell expansions, the title of the manuscript should be reconsidered.

Author response: We agree that the original title did not fully embrace the outlined strategy and the phenotypic assessment of NK cell differentiation. Suggested new title:

“Tracing Dynamic Expansion of Human NK Cell Subsets by High-Resolution Analysis of KIR Repertoires and Cellular Differentiation”

4) It would be much easier to follow text and figures, if the names of both, the clones and KIR genes recognized, would be consistently indicated throughout the manuscript (as depicted in Figure 1D-F, while it is missing in Figure 1A-C).

Author response: We agree and have changed the figure accordingly.

European Journal of Immunology

5) It is not clear how Figure S2 helps to identify clonally expanded populations, the reference in the text (pp3, line 20-24) does not clearly indicate what can be found in the figure. Moreover, the authors do not comment on Figure S2B and S2C. In line with comment I, impact and antibody conjugation should be discussed.

Author response: The text and Supplementary Figure 2 (Now Supplementary Figure 3) has been corrected to highlight the aspects of relevance for the current protocol, pertinent to the 2DS2 staining. For staining of KIR2DS5 and KIR3DS1 we refer to previously published strategies. See text on page 3.

6) On page 2, line 59, KIR2DL3 is written, but as indicated in Figure 2A and 2C, it should be KIR3DL1.

Author response: We apologize for this mistake. Now corrected.

Minor points:
7) In Figure 2B, it would be helpful to indicate the x-axis as in 2C and 2D

Author response: Agree, corrected.

8) The legend for supplementary figure 2A and D should mention the KIRotype plotted on the left side of the dot plots and how it was determined.

Author response: This figure has been revised as outlined above in response to comment 5. In the revised figure, the KIRotype is mentioned. The KIR typing protocol is described in the supplementary info with reference to the detailed description of the qKAT method.

Reviewer: 3

Comments to the Author
In this manuscript the authors describe an approach based on the combined use of various commercially available anti-KIR mAbs, using a 15-color flow cytometry, to characterize and quantify the different NK cell subpopulations based on their KIR expression. The described protocol includes different quality controls (QCs) that provide a flowchart allowing to exclude donors characterized by a KIR repertoire that gives rise to staining misinterpretation (e.g. false KIR2DL2/S2+ or KIR2DS1+ subsets). In particular, KIR2DL3*005 and KIR2DL3*015 have been previously described to have an atypical staining pattern. Indeed, both do not react with the anti-KIR2DL3 specific mAb (i.e. 180701) and KIR2DL3*005 reacts with the anti KIR2DL1/2DS1 mAb (i.e. EB6). QC1 evaluates the presence/absence of KIR2DL3*005. QC2 allows to identify subjects characterized by a cenA/cenA genotype (i.e. KIR2DL2/2DS2 negative) whereas QC3

European Journal of Immunology

allows to exclude donors that are KIR2DS1 positive. Correct and unambiguous interpretation of the stainings can be assessed only for the samples that pass all QCs. As also stated by the Authors, analysis of KIR genotype is always highly recommended, indeed still unrecognized exceptions of anti-KIR mAb reactivity can possibly exist.

The proposed strategy is clear, well defined and can provide great help for studies on the characterization of NK cells expressing one or another KIR. The correct assessment of the KIR phenotype is an excellent tool for additional analysis such as evaluation of clonal expansions of NK cells or identification of markers that can characterize the different NK cell subpopulations. The authors have a solid experience and good publications in this field.

Minor revision are requested:

1) Page 1 lines 40-41: the sentence should be modified including information on the impact of HLA class I (KIR-L) in KIR education.

Author response: We have included the terms education and KIR ligands in the brief sentence describing the interactions between KIRs and their corresponding HLA ligands (see page 1).

2) Page 2 lines 17-19: the sentence should be modified to better clarify that it is actually the presence of KIR2DL3*005, when co-expressed with KIR2DL2/S2 and/or KIR2DS1, that does not permit a further detailed analysis of the KIR repertoire.

Author response: Although this sentence in isolation becomes more clear with the change suggested by the reviewer it becomes very difficult to rephrase this particular sentence to the proposed active format since it would disrupt the flow and complicate reference to Figure 1E at the end. In response to this comment, we have chosen to rephrase the sentence coming earlier in the text: “These QCs allow for identification and exclusion of donors with KIR2DL3*005+ NK cells coexpressing KIR2DL2/S2+ and/or KIR2DS1+, since the latter KIRs cannot be distinguished from KIR2DL3*005 with currently available mAbs.” See changes in text on page 2.

3) A table summarising all mAb used for cytofluorimetric analysis is required and may be included in the Supplementary items. This table should indicate name of the clone, reactivity, source, type of labeling. Also the various steps of incubation are important to reproduce the method.

Author response: We have now included a table with the requested information. See also response to comment 1 of reviewer 1.

4) In the figures the + and – characters related to the various KIRs are too small and hardly visible (while these data are crucial). The use of a larger character is recommended.

European Journal of Immunology

Author response: Agree, changed accordingly.

5) Figure 1 panel D: the choice of representative donors with A/A and B/x haplotypes would be much more appropriate to show KIR2DS4 expression in A/A instead of B/x donor.

Author response: Good point! We have replaced the representative A/A and B/X donors.

6) Page 2 lines 56-59: The identification of KIR+ NK cells in relation with data shown in Fig. 2 is unclear or wrong: KIR2DL2/S2+ (and not only KIR2DS2+) NK cells in donor #018, KIR3DL1 (and not KIR2DL3) in donor #034.

Author response: Thanks for noticing these mistakes, which have been corrected. See changes in the text on page 2.

7) Fig. 2 B: There is no indication of the molecule in the abscissa of both dot plots (KIR2DL1 and CD57?)

Author response: Now corrected.

8) Fig. 2: (A) the NKG2A+NKG2C+ subset has not been considered; is it included in the NKG2A+ subset? Please, briefly comment this point.

Author response: Indeed, the double positive subset is included in the NKG2A+ subset analysis. In this cohort of 60 donors, the average frequency of the double positive subset was 1.6%. However, the point is well taken since the NKG2A+ expansion observed in donor #18 is in fact double positive. This is now discussed briefly in the paper on page 2 and in the figure legend on page 4.

9) The NKp30- CD57+ pattern has been considered as the hallmark of an expanded phenotype. Please provide the appropriate reference.

Author response: This comment is similar to comment 3 and 2 by reviewer 1 and 2, respectively. NKp30 was chosen as one key marker based on our previous examination of 204 healthy donors where it was found to be highly specific (Beziat et al, Blood 2013, Figure 5B). However, some donors harbor expansions with less clear staining patterns. For such expansions it may be necessary to extend the phenotypic panel and monitor additional differentiation markers. The basis for selecting NKp30 and CD57 and the potential weaknesses of the approach are now discussed on page 3.

10) The color code used to indicate the KIR combinations of the NK cells in each graph of figure 2 A would

help the reader. Y axes may be interrupted. This would shorten the height of the panels allowing the insertion of the color code.

Author response: In response to this comment we have moved Figure 2E to give room for the suggested change, which we think is excellent.

11) Supplementary Fig. 2: two different names for mAb recognizing 2DL5 are used: UP-R1 in the figure panel D, and 5.133 in the legend. 5.133 mAb is probably incorrect. Moreover, for the detection of KIR2DS5 positive cells the authors propose the use of NKVSF1 mAb, a mAb characterized by a broad reactivity with KIR2D. Why should the authors exclude KIR2DL5 positive cells from the analysis? Is there any evidence that NKVSF1 mAb recognizes also KIR2DL5?

Author response: We thank the reviewer for noticing these two important mistakes! Indeed, KIR2DL5 (detected by UP-R1) isn't recognized by NKVSF1. The currently used 16-color panel detects KIR2DS2 but not KIR3DS1 and KIR2DS5. Additional stainings are required to assess these KIRs. As pointed out by reviewer 2 (comment 5), the detailed description of combinations used to decode the expression of those KIRs is not an essential part of the algorithm for tracing adaptive-like NK cell responses. Therefore, the text and supplementary Figure 2 (Now Supplementary Figure 3) has been revised to highlight the aspects of relevance for the current protocol, pertinent to the 2DS2 staining. For staining of KIR2DS5 and KIR3DS1 we refer to previously published strategies. See revised text on page 3.

12) A very recent paper (Della Chiesa et al. J Immunol 2014) described the expansion of NK cells expressing activating KIRs in NKG2C-/- patients experiencing CMV infection. This reference is relevant and should be added.

Author response: Agree, reference added.

Second Editorial Decision – 27 March 2014

Dear Dr. Malmberg,

It is a pleasure to provisionally accept your manuscript entitled "Tracing dynamic expansion of NK cell subsets by high resolution analysis of killer cell immunoglobulin-like receptor repertoires and cellular differentiation" for publication in the European Journal of Immunology. For final acceptance, please follow the instructions below and return the requested items as soon as possible as we cannot process your manuscript further until all items listed below are dealt with.

Please note that EJI articles are now published online a few days after final acceptance (see Accepted Articles: http://onlinelibrary.wiley.com/journal/10.1002/(ISSN)1521-4141/accepted). The files used for the Accepted Articles are the final files and information supplied by you in Manuscript Central. You should

therefore check that all the information (including author names) is correct as changes will NOT be permitted until the proofs stage.

We look forward to hearing from you and thank you for submitting your manuscript to the European Journal of Immunology.

Yours sincerely,
Laura Soto Vazquez

on behalf of
Prof. Marco Colonna

Editorial Office
European Journal of Immunology
e-mail: ejied@wiley.com
www.eji-journal.eu
